# HIV-1 resistance mutations and genetic diversity among children failing antiretroviral treatment in five healthcare facilities in Benin, West Africa

**DOI:** 10.1371/journal.pone.0317882

**Published:** 2025-01-29

**Authors:** Edwige Hermione Dagba Gbessin, Edmond Tchiakpe, René Kpemahouton Keke, Nicole Vidal, Michel Kiréopori Gomgnimbou, Haziz Sina, Euloge Senan Adjou, Aldric Afangnihoun, Moussa Bachabi, Akadiri Yessoufou, Abdoul-Salam Ouedraogo, Lamine Baba-Moussa

**Affiliations:** 1 NAZI BONI University, Bobo-Dioulasso, Burkina Faso; 2 National Reference Laboratory of Health Program Fighting Against AIDS in Benin (LNR/PSLS), Health Ministry, Akpakpa, Littoral, Benin; 3 Faculty of Sciences and Technology (FAST) and Institute of Applied Biomedical Sciences (ISBA), Department of Biochemistry and Cellular Biology, Laboratory of Cell Biology and Physiology, University of Abomey-Calavi, Abomey-Calavi, Atlantic, Benin; 4 UMI233 -TransVIHMI, Université de Montpellier, Institut National de la Santé et de la Recherche Médicale (INSERM), Institut de Recherche pour le Développement (IRD), Montpellier, France; 5 Higher Institute of Health Sciences (INSSA), Nazi Boni University, Bobo-Dioulasso. Molecular Biology Laboratory, Muraz Center, Bobo-Dioulasso, Burkina Faso; 6 Faculty of Sciences and Technology (FAST), Laboratory of Biology and Molecular Typing in Microbiology (LBTMM), University of Abomey-Calavi, Atlantic, Benin; 7 Health Program Fighting Against AIDS in Benin (PSLS), Health Ministry, Akpakpa, Littoral, Benin; 8 Laboratory of Emerging and Re-emerging Pathogens (LaPathER), Doctoral School of Health Sciences, Nazi BONI University, Bobo-Dioulasso, Burkina Faso; University of Cincinnati College of Medicine, UNITED STATES OF AMERICA

## Abstract

**Background:**

Antiretroviral treatment increases the risk of accumulation of resistance mutations that negatively impact the possibilities of future treatment. This study aimed to present the frequency of HIV-1 antiretroviral resistance mutations and the genetic diversity among children with virological failure in five pediatric care facilities in Benin.

**Methods:**

A cross-sectional study was carried out from November 20, 2020, to November 30, 2022, in children under 15 years of age who failed ongoing antiretroviral treatment at five facilities care in Benin (VL > 3log_10_ on two consecutive realizations three months apart). Viral loads were measured using the m2000 RealTime Abbott platform. Genotyping was carried out with the commercial Viroseq kit. Sequences were read on the ABI 3500 sequencer and then edited with ViroSeqHIVv3.0 software. The HIV drug resistance database at Stanford University was used to identify mutations and viral subtypes were assigned by phylogenetic analyses.

**Results:**

The HIV-1 pol gene was sequenced in 47 participants with virological failure of antiretroviral treatment. The median age was 120 [Interquartile Range 90–144] months. The prevalent treatment was EFV base regimen (22/47; 46.8%). Median viral load was 4.39 log_10_ [IQR 3.81–4.86 log_10_] respectively. Resistance testing was successful among (37/47; 78.72%) children, resistance mutations were detected in (32/37; 86.48%) children, and (29/32; 90.62%) had at least one surveillance drug resistance mutation. Respectively (25/32; 78.12%), (28/32; 87.5%), (4/32; 12.90%), (22/32; 68.75%) had at least one resistance mutation associated with NRTIs, NNRTIs, PIs and NNRTIs+NRTIs. (12/32; 37.5%) of children carried mutations related to TAMs. the most frequently NRTIs identified were M184V (21/62; 33.9%) followed by TAMs (20/62; 32.2%) and T69G/D (2/62; 3.2%)s. Among mutations associated with NNRTIs K103N represented (18/64; 28.1%) followed by P225H (7/64; 10.9%). The I54V (3/6; 50%) mutation is the major PI observed.

Genetic diversity is characterized by a preponderance of CRF02_AG (72%, 23/32), followed by unique recombinant forms (URFs) (25%, 8/32) and one subtype G.

**Conclusion:**

A high rate of mutations has been observed in children. These data underline the importance of implementing routine genotypic testing in the biological monitoring of infected children to anticipate the accumulation of resistance mutations and thus compromise the treatment options available in Benin.

## Introduction

The issue of antiretroviral resistance in HIV-infected children is still topical, particularly because of its impact on the mortality and morbidity associated with this infection in this segment of the population [1]. At the end of 2022, of the 1.5 million children living with HIV worldwide, 57% had access to treatment [2]. Despite several advancements, it may be difficult to achieve the second UNAIDS target, which stipulates that 95% of HIV-infected people should be on antiretroviral treatment [3,4]. The widespread use of antiretroviral therapy (ART), particularly programs to prevent mother-to-child transmission has led to a 58% reduction in new infections among children. However, suboptimal antiretroviral therapy in mothers, antiretroviral prophylaxis and difficulties in administering treatment in newborns strongly contribute to the development of treatment resistance especially in the African context [5,6]. This selection of resistance mutations inevitably leads to the failure of antiretroviral therapy [7]. Regular follow-up of these children by quantifying the viral load makes it possible to anticipate virological failure and avoid an accumulation of resistance mutations [8].

HIV prevalence in the general population is 0.8% in Benin [9,10]. The ART program started in 2005 in the country and according to the 2021 monitoring report, around 2,392 children are living with HIV, of whom 2,383 are on antiretroviral treatment[11]. According to the national policy, ART must be initiated in all children living with HIV, regardless of T cell count (CD4+T cells) and clinical stage. Considerable progress has been made in biological and therapeutic care. Indeed, in terms of biological care, the operational capacity of laboratories has been increased through the acquisition and provision of molecular biology equipment for the realization of viral load and genotyping. This strategy has made it possible to increase national coverage by offering these services to the greatest number of patients. Therapeutic management has been improved thanks to certain actions, including introducing more effective and better-tolerated molecules such as Dolutegravir in therapeutic regimens, organizing consultation workshops, and sharing experiences and good practices of the actors involved (prescribing doctors or nurses). According to the national guidelines for the therapeutic management of HIV infection and contained in the document of national HIV standards and policies, the treatment regimens in force in Benin for the treatment of children are made up of 2NRTI+1NNRTI/PI/INSTI. Thus several combinations are possible, namely: ((ABC/ AZT) + 3TC + (DTG/ LPV/r/ EFV)) (Abacavir/Zidovudine + Lamivudine + Dolutegravir/lopinavir boosted by Ritonavir/ Efavirenz) or ((TDF/ AZT) + 3TC + (DTG/ EFV)) (Tenofovir/Zidovudine + Lamivudine + Dolutegravir/Efavirenz). Those therapeutic lines are based on the child’s weight which is less than or more than 30 kg without forgetting iron supplementation when using DTG [12]. Biological monitoring of the effectiveness of antiretroviral therapy in a resource-limited country context is mainly based on TCD4+ lymphocyte counts and plasma viral load quantification every 6 months [12]. The challenge in pediatric care in our country remains the maintenance of efficient ART despite the difficulties of compliance and the emergence of drug resistance described. The issue of pediatric antiretroviral resistance in Benin remains alarmingly underexplored, exposing a gap that needs to be addressed. However, a study conducted in 2018 at the pediatric clinic of the National University Hospital Center in Cotonou revealed a high frequency of resistance to at least one antiretroviral in children on both first-line (39/45; 87%) and second-line (7/8; 88%) regimens [13]. Since this study, efforts have been made in the training of doctors and psychologists responsible for the care of infected children under ART in Benin. Surveillance of antiretroviral resistance is important not only to guide treatment guidelines but also to explore the role of routine genotypic testing in the biological monitoring of children.

This study was conducted in five pediatric facilities care in Benin and has the main objective of presenting the frequency of HIV-1 resistance mutations associated with ART and genetic diversity in children with virological failure.

## Methods

### Study population

A cross-sectional study was carried out from November 20, 2020, to November 30, 2022, in children under 15 years of age who had ART failed (VL > 3log_10_) at five facilities care in the country: Abomey-Calavi Hospital, a non-governmental organization (NGO) Racines, Bethesda Hospital, University Hospital Center for Mothers and Children (CHU-MEL) and Suru-Léré Hospital sites. The recruitment of participants was carried out from November 20, 2020, to October 15, 2021. The sequencing, data collection and analysis were done from October 20, 2021 to November 30, 2022.

### Blood samples

Whole blood samples were collected in EDTA tubes from all eligible participants. Plasma aliquots were taken and stored at -80°C.

### Viral load testing

Plasma HIV-1 RNA viral load (VL) was performed using Abbott RealTime HIV-1 assay (Abbott Molecular, Inc) in the National Reference Laboratory Fighting Against AIDS in Benin (NRL/PSLS). The linear range of 40–10.000.000 copies/ml with a detection limit of 40 copies/ml was defined by the manufacturers [14].

### Drug resistance genotyping

PCR and genotyping of HIV-1 drug resistance were performed using the Viroseq HIV-1 v2.0 Genotyping Kit, as previously described according to the manufacturer’s instructions [15]. This kit is a complete package of the necessary reagents from RNA extraction to sequencing reactions. This technique amplifies the entire (amino acids 1–99) of the protease (Pr) gene and 2/3 (amino acid 1–335) of the reverse transcriptase (RT) using seven primers (A, B, C, D, F, G, H) to generate a 1.8kb PCR product. This amplicon was used as a sequencing template to generate approximately 1.3 kb of consensus sequence. This pol gene region of the HIV-1 fragments was subjected to Applied Biosystems’ ABI 3500 genetic analyser. The consensus sequences were assembled and analyzed by the Viroseq HIV-1 V2.5 377 software to identify mutations compared to an HXB2 reference sequence of the B subtype (GenBank accession number K03455).

Genotypic resistance profiles were obtained by submitting the sequences to the HIV drug resistance database at Stanford University (https://hivdb.stanford.edu/).

### Phylogenetic analysis

Nucleotide sequences were aligned with a set of reference sequences representative of HIV-1 group M subtypes and Circulating Recombinant Forms (CRFs) in West and Central Africa with three reference sequences per subtype/CRF. Sequences were aligned using MAFFT version 7 (https://mafft.cbrc.jp/alignment/server/). Gap positions were removed under AliView. Each sample sequence was submitted a similarity plot analysis with SimPlot v3.5.1 from the alignment [16]. For recombinant profiles, individual bootscan analyses were performed using the Kimura two-parameter model with a window of 350 bp advancing in 20 bp increments, and the alignment was cut into several segments as defined by the bootscan plot. Each segment was submitted to a Maximum Likelihood (ML) phylogenetic analysis to confirm the subtype/CRF designation [17]. The final alignment was drawn keeping all HIV-1/M subtypes and the sub-subtypes and CRFs within the new sample sequences. The final tree was drawn under GTR+F+R4 as the best-fit model of nucleotide substitution according to BIC and 1000 bootstrap resampling, by using IQ-Tree v1.6.12 server (http://iqtree.cibiv.univie.ac.at). The consensus tree was edited with FigTree v1.4.4.

Recent transmission clusters were ascertained based on short branch lengths (≤ 0.015) and high support values (>98%).

The newly generated sequences were submitted to Genbank under accession numbers: OR139618 to OR139649.

### Statistical analysis

Data on sociodemographic characteristics and treatment history were collected from participants’ medical records using a questionnaire. All data were entered into Excel 2016 and analyzed with this software. A descriptive analysis was carried out, with data presented in the form of numbers, percentages, mean, interquartile range, and median with their 95% confidence intervals.

### Ethics approval and confidentiality

The protocol was also approved by the National Ethics Committee for Health Research (CNERS) under trial number 30 of August 30, 2020 (Reference N°: 111/MS/DRFMT/CNERS/SA).

Written informed consent was obtained from the parents or legal guardians of the participants involved in the study. Confidentiality and anonymity of the information was also maintained. The study was conducted using the relevant guidelines and regulations.

## Results

### Patient characteristics

Forty-seven cases with a viral load greater than 3log_10_ copies/ml were included in this study for genotyping. Their median age was 120 [Interquartile Range 90–144] months and they were predominantly female (26/47; 55.3%). The most common treatment used was a regimen based on EFV (22/47; 46.8%). The mean CD4+T cells count was 760.4 with a median of 603 [Interquartile Range 366.5–1021.5] cells/μl blood. The mean viral load was 4.43 log_10_ and, the median was 4.39 [Interquartile Range 3.81–4.86 log_10_] copies/ml. Table 1 describes the demographic characteristics, biological data (VL), and DRMs among children included in the study.

**Table 1:** Demographic characteristics, biological data (VL) and DRM among children included in the study.

Variables	N
**Sample size**	**47**
**Sex (%)**	
**M**	21 (44.68)
**F**	26 (55.32)
**Median Age [Interquartile range] months**	120 [90–144]
**Participants distribution according to facilities care (N DRMs / N virologic failure)**	
**Suru-Léré (public hospital)**	09 /13
**Abomey-Calavi (public hospital)**	07 / 13
**CHU-MEL (public hospital)**	06 / 11
**Bethesda (private hospital)**	06 / 07
**Racines (Non-Governmental Organisation)**	01 / 03
**Treatment (%)**	
**(AZT/TDF/ABC)+3TC+LPV/r**	19 (40.42)
**(AZT/ABC/TDF)+3TC+EFV**	22 (46.8)
**AZT+3TC+NVP**	3 (6.4)
**(TDF/ABC/AZT)+3TC+DTG**	5 (10.6)
**TDF+3TC+ATZ/r**	1 (2.1)
**Median defined time on ART (months)**	54
**< 12**	02
**12–23**	01
**≥ 24 ≤ 144**	27
**ND**	17
**CD4+ T cells (cells/μl)**	
**< 100**	02
**100–200**	01
**≥ 200 ≤ 1501**	44
**Median CD4 count [Interquartile range]**	603 [366–1021] cells/μl
**Median viral load [Interquartile range]**	4.39 [3.81–4.86] log_10_
**Genotyped (%)**	37 (78.72)
**DRMs (%)**	32 (86.48)
**NRTIs**	25 (78.12)
**NNRTIs**	28 (87.5)
**Pis**	04 (12.90)
**NRTIs + NNRTIs**	22 (68.75)
**NRTIs+NNRTIs+Pis**	02 (6.25)
**At least one SDRM**	29 (90.62)

ABC: Abacavir, 3TC: Lamivudine, LPV: Lopinavir, AZT: Zidovudine, TDF: Tenofovir, EFV: Efavirenz, NVP: Névirapine, DTG: Dolutegravir, ATZ: Atazanavir, ND: Not defined, ART: Antiretroviral treatment, CD: Cluster of differentiation, IQR: Interquartile, DRM: Drug resistance mutations, NRTI: Nucleoside reverse transcriptase inhibitors, NNRTI: Non nucleoside reverse transcriptase inhibitors, PI: Protease inhibitors.

### Resistance mutations

Sequencing of HIV-1 gene *Pol* was successful among (37/47; 78.72%) children. Resistance mutations were detected in (32/37; 86.48%) and (29/32; 90.62%) had at least one surveillance drug resistance associated mutation. Of these, approximately (25/32; 78.12%), (28/32; 87.5%), (4/32; 12.90%), (22/32; 68.75%), (02/32; 6.25%) had at least one resistance mutation associated with NRTIs, NNRTIs, PIs, NNRTIs+NRTIs, and NNRTIs+NRTIs+PIs respectively. (12/32; 37.5%) of children carried mutations associated with TAMs (Thymidine Analog Mutations) including TAMsI (M41L, L210W, and T215Y) and TAMsII (D67N, K70R, T215F, and K219Q/E).

**Fig 1:**
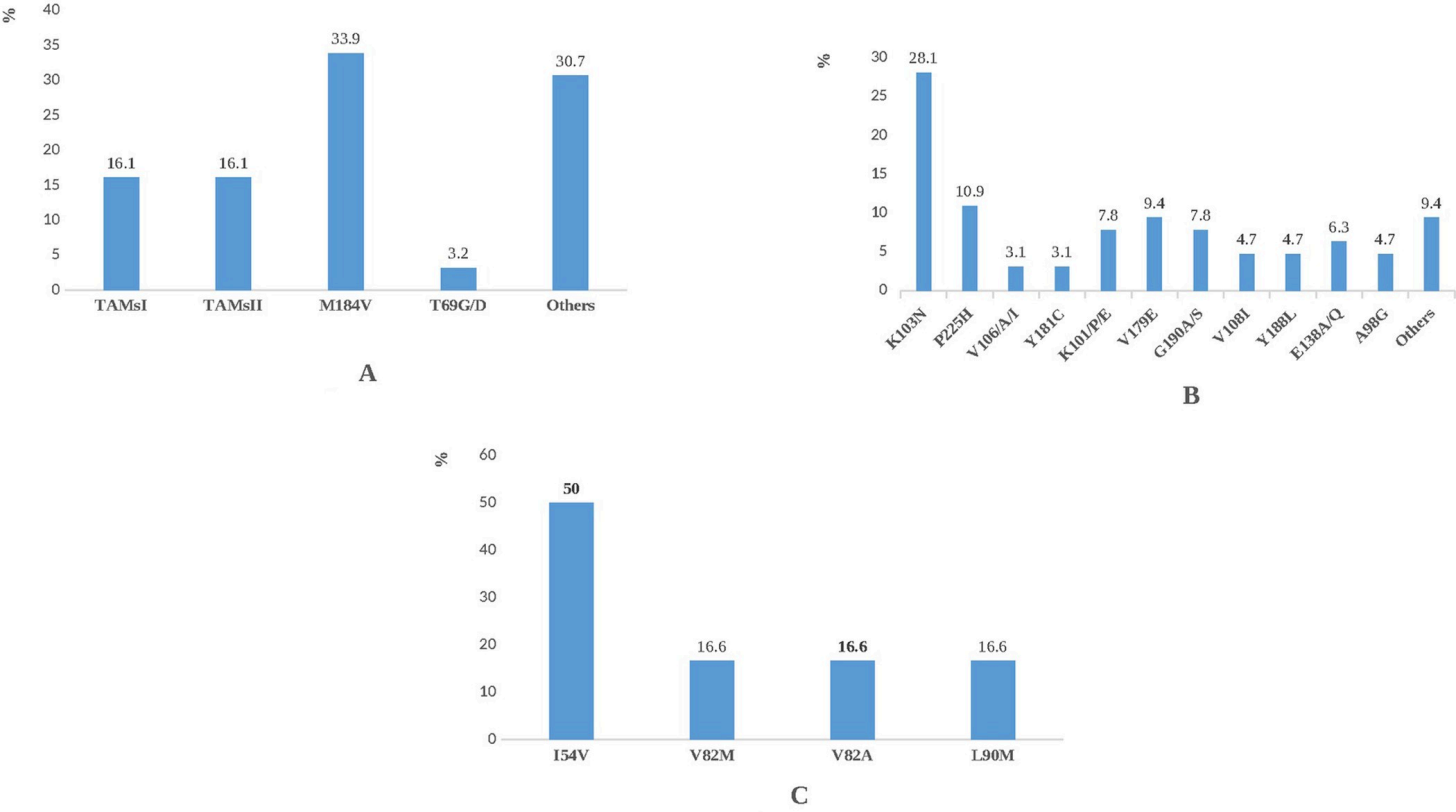
A) NRTIs resistance associated mutation prevalence, B) NNRTIs resistance associated mutations prevalence, C) PIs resistance associated mutations prevalence.

Among mutations associated to NRTIs, TAMs represented (20/62; 32.2%) followed by M184V (21/62; 33.9%) and T69G/D (2/62; 3.2%). Others NRTIs associated mutations were present (19/62; 30.7%) (Fig 1A). K103N represents (18/64; 28.1%) among mutations associated with NNRTIs followed by P225H (7/64; 10.9%). Other NNRTIs encountered are presented in Fig 1B.

The major PIs associated mutation, I54V (3/6; 50%) observed is presented in Fig 1C. Some PIs accessory mutations were observed, such as K20T, L89IV and Q58E (respectively 1/3; 33.33%).

### Distribution of 32 HIV‑1 group M variants among children in Benin

A predominance of CRF02_AG (72%, 23/32) was observed in this population, followed by unique recombinant forms (URFs) (25%, 8/32). One sequence harbored pure subtype G (3.12%, 1/32). Among unique recombinants, two sequences (4183 and 13193) were segregated into one recent transmission chain.

**Fig 2:**
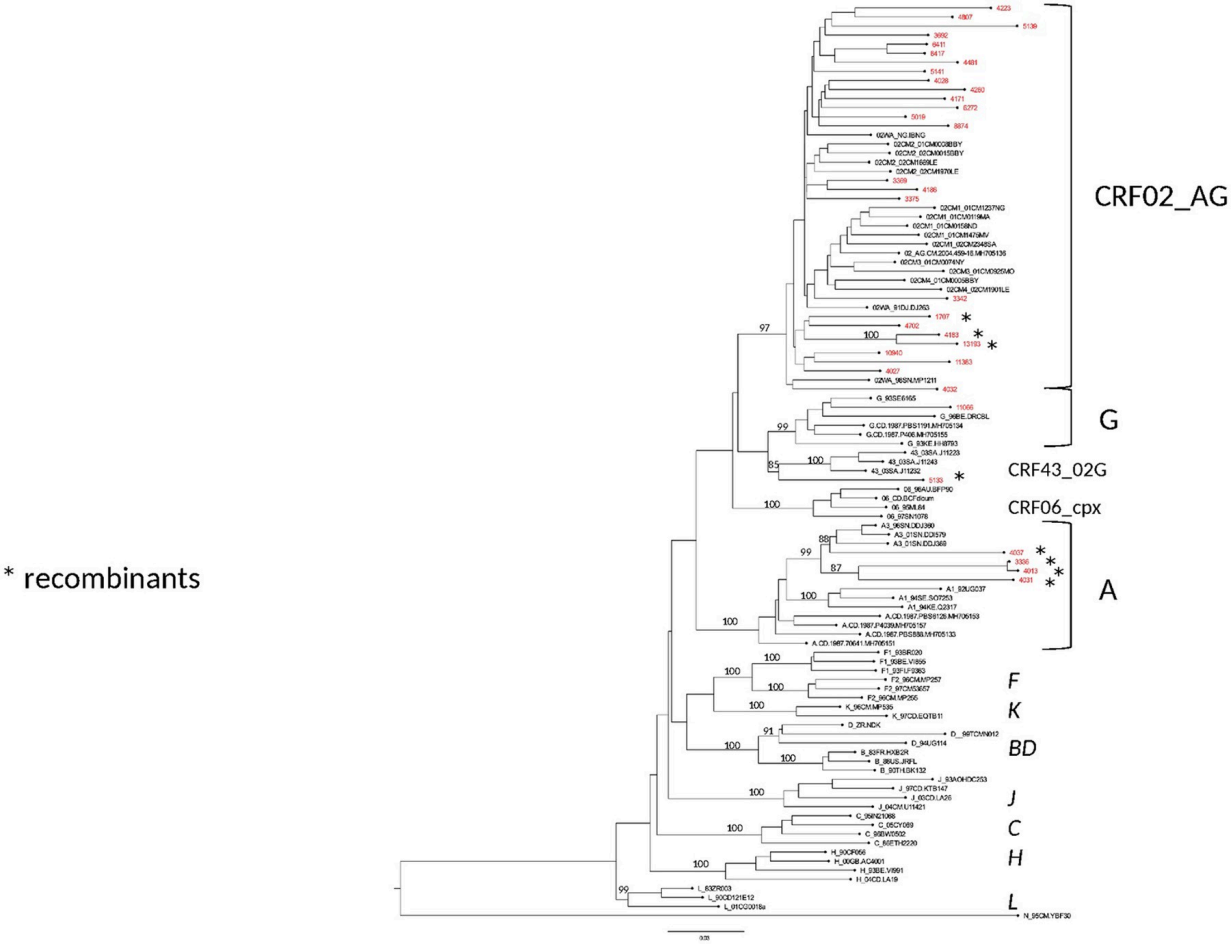
Phylogenetic relationships of the study sequences with reference HIV-1 genomes.

The phylogenetic tree is presented in Fig 2.

Phylogeny was inferred under IQ-Tree version 1.6.12 by maximum likelihood for 1302 unambiguously aligned nucleotide sequences of *protease* and partial *reverse transcriptase* genes for 32 samples from the study with 1000 bootstrap resampling under GTR+F+R4 as the best-fit model of nucleotide substitution according to BIC. The consensus tree was edited with FigTree v1.4.4. HIV-1 reference sequences are in black, the new sequences from the study are in red. Branch supports are indicated for the main clades. Asterisks refer to unique recombinant strains.

**Fig 3:**
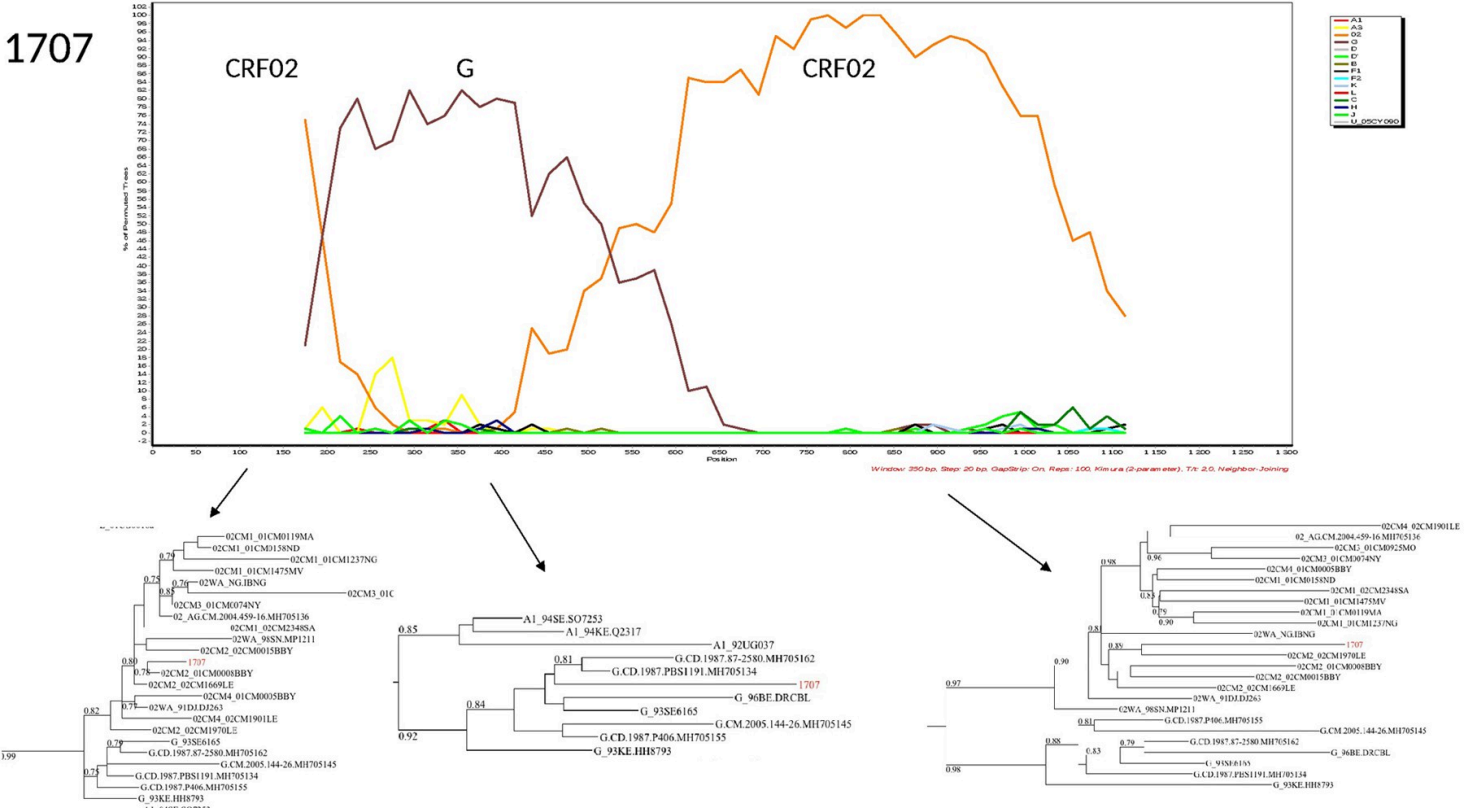
Bootscans of genome 1707, Unique recombinant forms identified of the study sequences. The bootscan analysis was performed using SimPlot software with a 350 bp window and a 20 bp step. The x-axis represents the position of the nucleotides in alignment. The different clades of HIV-1 are represented by specific color codes.

**Fig 4:**
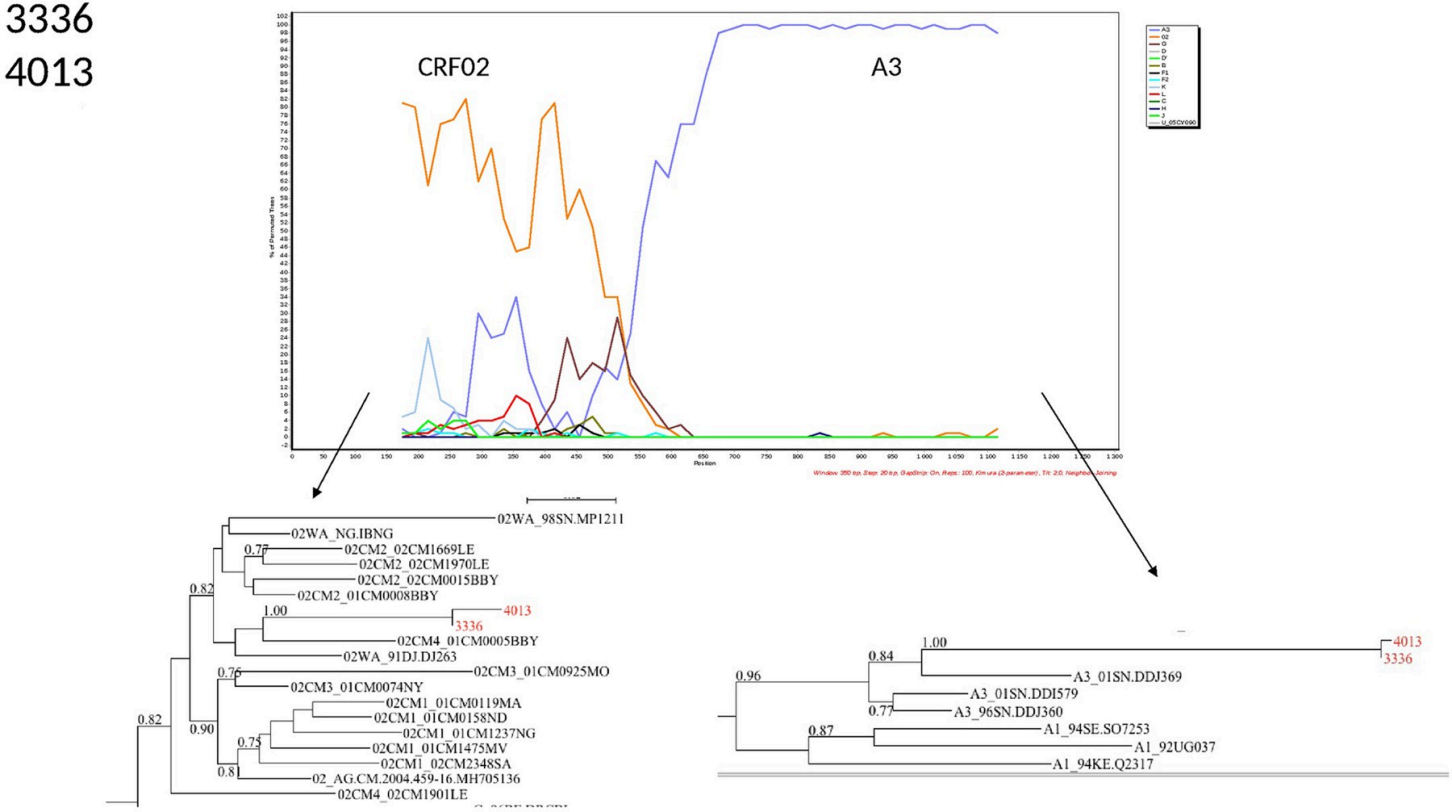
Bootscans of genome 3336 and 4013, Unique recombinant forms identified of the study sequences. The bootscan analysis was performed using SimPlot software with a 350 bp window and a 20 bp step. The x-axis represents the position of the nucleotides in alignment. The different clades of HIV-1 are represented by specific color codes.

**Fig 5:**
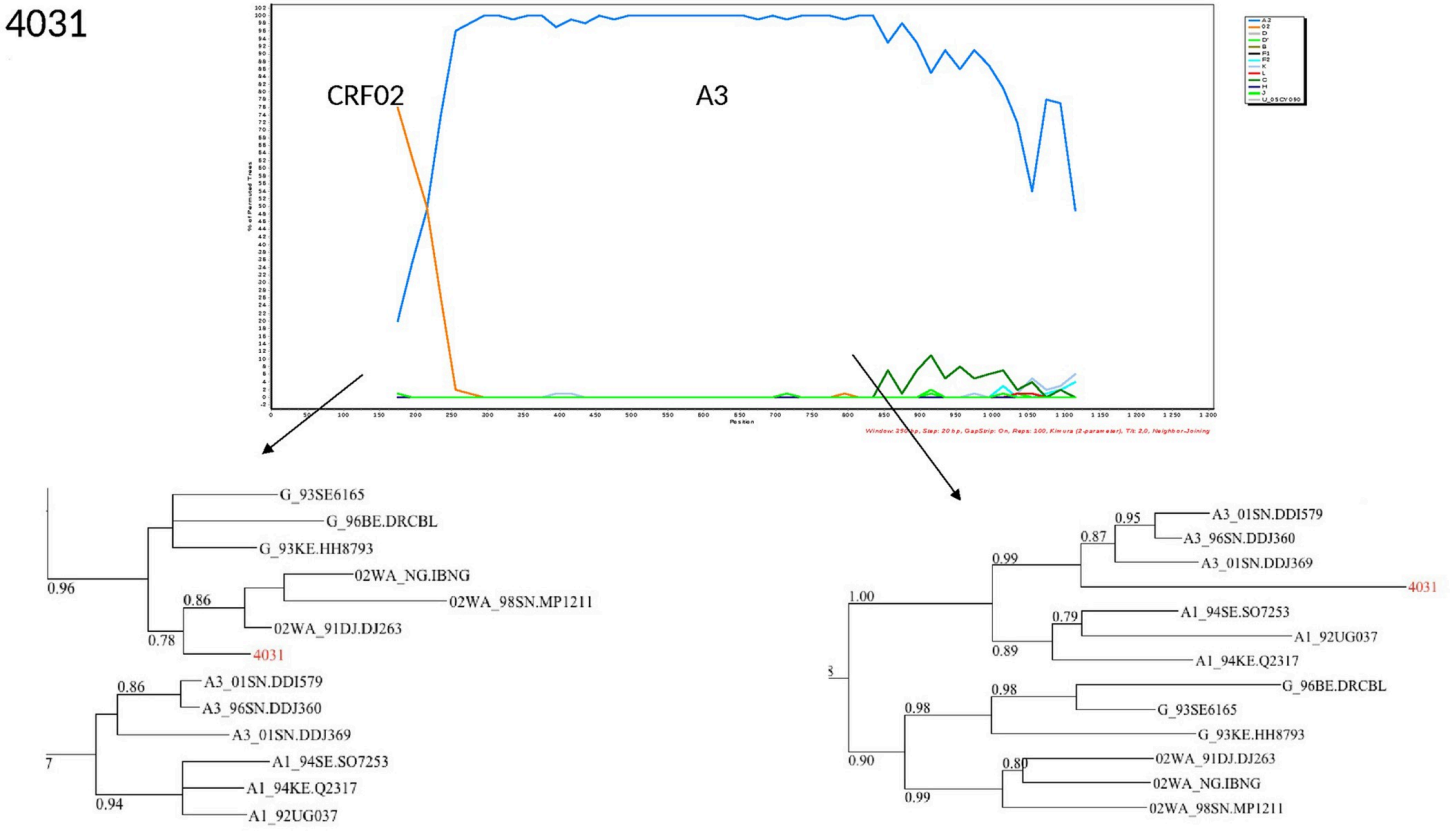
Bootscans of genome 4031, Unique recombinant forms identified of the study sequences. The bootscan analysis was performed using SimPlot software with a 350 bp window and a 20 bp step. The x-axis represents the position of the nucleotides in alignment. The different clades of HIV-1 are represented by specific color codes.

**Fig 6:**
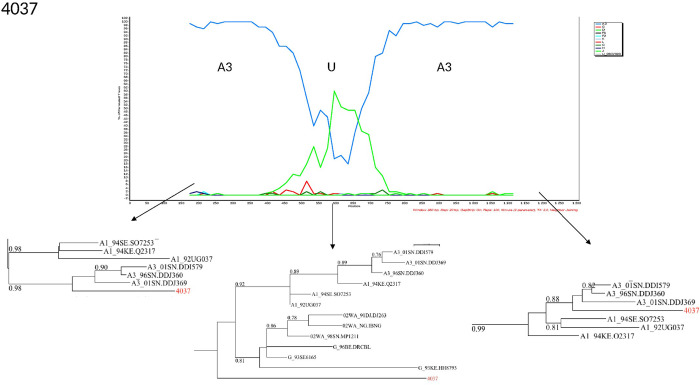
Bootscans of genome 4037, Unique recombinant forms identified of the study sequences. The bootscan analysis was performed using SimPlot software with a 350 bp window and a 20 bp step. The x-axis represents the position of the nucleotides in alignment. The different clades of HIV-1 are represented by specific color codes.

**Fig 7:**
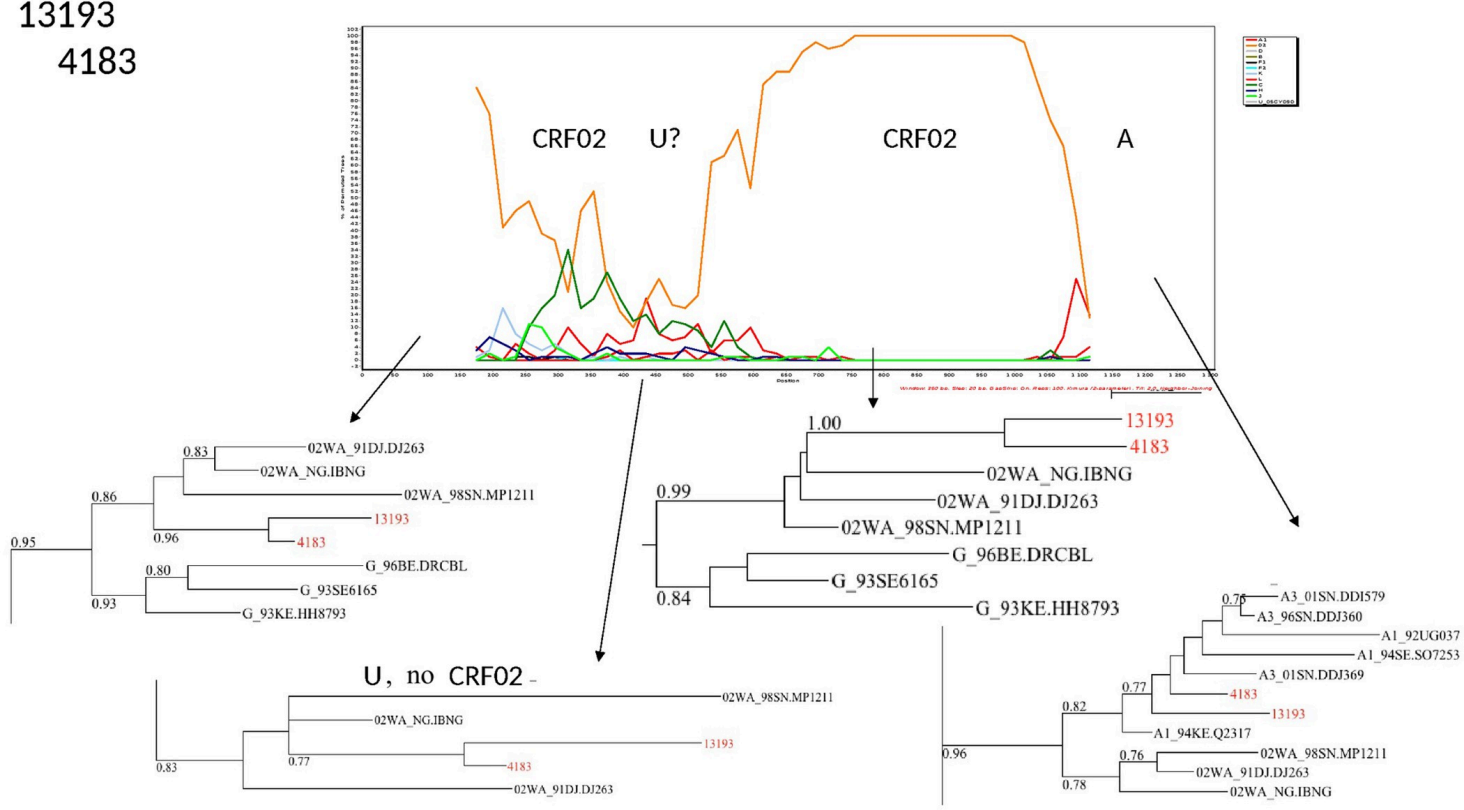
Bootscans of genome 4081 and 13193, Unique recombinant forms identified of the study sequences. The bootscan analysis was performed using SimPlot software with a 350 bp window and a 20 bp step. The x-axis represents the position of the nucleotides in alignment. The different clades of HIV-1 are represented by specific color codes.

**Fig 8:**
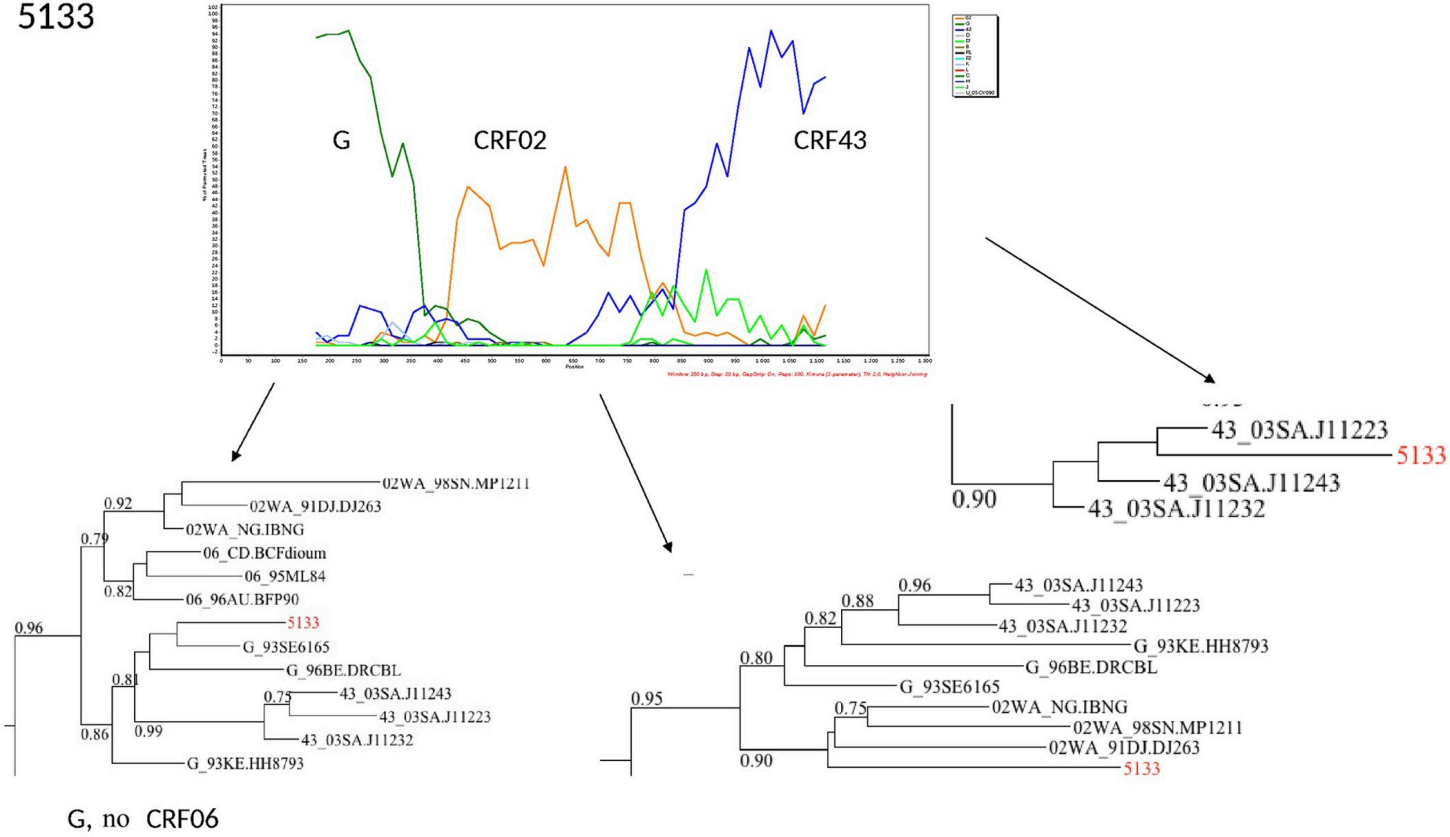
Bootscans of genome 5133, Unique recombinant forms identified of the study sequences. The bootscan analysis was performed using SimPlot software with a 350 bp window and a 20 bp step. The x-axis represents the position of the nucleotides in alignment. The different clades of HIV-1 are represented by specific color codes.

The characterization of the recombinants by bootscanning allowed us to identify the sequences of three samples as CFR02_AG/A3, two as CRF02/A, one CRF02_AG/G/CRF02_AG, one A3/U/A3, and one G/CRF02/CRF43. The bootscans of recombinant forms are presented in Figs 3–8.

## Discussion

This study reported the frequency of antiretroviral resistance in a population of children with virological failure and followed up at five treatment sites in Benin. Most participants did not have an immune deficiency as defined by the WHO pediatric antiretroviral treatment guidelines (median CD4 count of 603 cells/μl). Viral loads, although high with a median of 4.39 log_10_ (24,547 copies/ml), were not able to obtain optimal performance during sequencing because there was about 21.28% PCR failure. Knowing that the detection sensitivity of the Viroseq technique is 2,000 copies/ml, the sequencing failure rate in the present study can be partly justified by the fact that approximately 12.77% of the participants had a viral load less than 2,000 copies/ml (3.30log_10_). In addition, sequencing failures related to the Viroseq technique have been reported in the literature. Indeed, it would seem that the performance of this technique is altered by the sequence variations of the non-B subtypes [18–20]. The Viroseq technique was developed for subtype B, which circulates mainly in northern countries, but the predominant subtype in West Africa is the non-B subtype.

The frequency of antiretroviral resistance among participants in this study was 86.48% with 87.5%, 78.12%, and 12.90% mutations in NNRTIs, NRTIs and PIs, respectively. The frequency of dual-class mutations was significant (68.75%) and 02 participants harboured triple-class mutations. These high rates of mutations observed could be justified by the relatively long duration of antiretroviral use in the participants in this study, which averaged 54 months. Although the size of the samples differs. The results are superimposed on those obtained at the Hubert Maga University Hospital Center which was called National University Hospital Center in 2018 which reported 71%, 84%, and 65% resistance to NNRTI and double-class NRTIs respectively [13]. However, no resistance to PIs was found in the study in children on first-line treatment. This difference in PI resistance in the two studies may be explained by the larger number of participants on PI treatment in the present study (20 versus 12). Similar frequencies have also been reported in some African countries such as Senegal [21], Ethiopia [22], Tanzania [23] and Rwanda [24]. The high frequency of dual-class resistance observed in participants in this study strongly compromises future treatment options in these participants, particularly in the two children who harboured triple-class resistance NRTI+NNRTI+PI. Children who are likely to have been exposed to antiretrovirals used in the prevention of mother-to-child transmission (PMTCT) program. It will therefore be necessary to explore and implement other combinations that are more efficient for these participants.

As in most studies conducted in Africa and elsewhere, the M184V mutation is the most prevalent in the NRTI class [25–27]. This mutation confers high-level resistance to Lamivudine and Emtricitabine but also low/intermediate resistance to Abacavir. It should be noted, however, that this same mutation increases susceptibility to Zidovudine and Tenofovir [28,29]. Among the participants, 37.5% (12/32) selected TAMs with as many type I as type II TAMs. Similar results have been reported in Cameroon [30]. These mutations greatly reduce viral susceptibility to zidovudine [31]. In this case, the selection for this mutation is probably due to the high use of zidovudine in the prevention of mother-to-child transmission program. On the other hand, it has been reported that in the case of subtype C, the combination of M184V and TAMs significantly increased susceptibility to Zidovudine [32].

In the NNRTI class, there is a prevalence of K103N (18/64, 28.1%) and P225H (7/64, 10.9%) which confer resistance to nevirapine and Efavirenz and therefore compromises the use of these two drugs in these participants. Indeed, these mutations are selected in the event of exposure to these two molecules or the event of suboptimal treatment due to poor compliance [33,34]. Similar results were obtained in other contexts [23,35]. In addition, NNRTI-associated V106A, Y188L, and P225H mutations confer cross-resistance to Rilpivirine, Etravirine, and Doravirine [36]. These molecules have never been used in treatment protocols in Benin.

PI mutations were observed in four participants, two of whom had intermediate resistance to Atazanavir and the remaining two had low-level resistance to the same drug. As this drug is used in adult alternative treatment lines, the presence of this mutation suggests the mother’s transmitted resistance. The most common mutation found in participants is I54V, a polymorphic mutation that reduces susceptibility to almost all PIs except boosted Darunavir (DRV/r). The gradual replacement of NRTIs with integrase inhibitors (INSTIs) in treatment protocols in Benin is expected to improve the management of these participants.

The genetic diversity in the population studied is characterized by the pure G subtype (3.12%, 1/32) and a predominance of recombinant forms with the most preponderant CRF02_AG (72%, 23/32). CRF02_AG is the most isolated recombinant form in West Africa [37]. This finding is corroborated by observations made at the pediatric clinic of the HKM University Hospital in Cotonou and in treatment-naïve adults [11,38]. The errors of RT and the high replication capacity of HIV generate very diverse viral variants, sometimes even within the same individual [39]. This multitude of viral variants is responsible for the scale of the epidemic and the complexity of management. Among them, the recombinant forms of UFRs and CRFs in particular represent the vast majority of the genetic diversity of HIV [40]. Among the UFRs identified in a non-negligible proportion (25%, 8/32), several derive from clades that circulate in the West African sub-region: Senegal, South Africa, Nigeria, and Cameroon as indicated by the different phylogenetic trees. The presence of URFs suggests that children were born to mothers infected with more than one virus strain as previously described in the country [41].

The presence of the recombinant G/CRF02/CRF43 could be explained by migratory flows linked to trade between Saudi Arabia and Benin, but the border proximity with Nigeria seems more likely because recombinant forms have been identified in a higher proportion (16%) in this country. Indeed, although the recombinant CRF43_02G was described for the first time in Saudi Arabia, estimates of its appearance in Nigeria date back to 1971 (95% HPD: 1952–1983) [42]. The recombinant CRF02_AG/A3 has been previously isolated from Senegal and is associated with a faster progression to the AIDS stage and a high proportion of death [43,44]. The A3 sub-subtype is circulating in some neighbouring countries such as Senegal, Niger, Nigeria, and Ivory Coast [45]. The recombinants identified in this study could come from the mosaic of variants previously described in Benin and the sub-region [46]. Regarding the transmission cluster, there does not seem to be any epidemiological data suggesting they have family ties. Indeed, although these two samples were obtained in the same department, these participants were enrolled in different health facilities care. For the confidentiality of data relating to the participants’ informed consent, we could not request additional information to explain this transmission cluster.

### Limitations

The major limitation of our study is the small sample size. Another important limitation is the unavailability of in-house genotyping kits to retest samples that have failed genotyping. In addition, it would also have been interesting to investigate the two sequences resulting from a recent chain of transmission. Also, it would be desirable to evaluate the prevalence of resistance to antiretroviral, particularly to integrase strand transfer inhibitors, in a larger cohort of children. In the current context of widespread use of this therapeutic class, monitoring resistance is proving to be a necessity to help programs ensure optimal treatment for all.

## Conclusion

The prevalence of antiretroviral resistance is high in the study population, highlighting the importance of switching to antiretroviral with a high genetic barrier, such as Dolutegravir, in pediatric treatment. The generalization of the use of integrase strand transfer inhibitors is a solution to which prevention and resistance monitoring actions must be associated as recommended by the WHO to guarantee a better quality of life for children under treatment.

## Supporting information

S1 File (XLSX)

S1 Questionnaire (DOCX)

## Abbreviations

ART: Antiretroviral Treatment
CD4: Cluster of differentiation
CRF: Circulating Recombinant forms
DBS: Dried blood spots
DRM: Drug Resistance Mutations
ECOWAS: Economic Community of West African States
EDTA: Ethylene diamine tetraacetic acid
HIV: Human Immunodeficiency Virus
IQR: Interquartile
LNR/PSLS: Reference National Laboratory Fighting Against AIDS in Benin NRTI: Nucleoside reverse transcriptase inhibitors
NNRTI: Non-Nucleoside reverse transcriptase inhibitors
INSTI: Integrase Strand Transfer Inhibitor
PCR: Polymerase Chain Reaction
PI: Protease inhibitors
PLHIV: People living with HIV
RNA: Ribonucleic Acid
TAM: Thymidine Analog Mutations
URF: Unique Recombinant forms
VL: Viral load
WHO: World Health Organization
