# Supplementary material for: HIV-1 resistance mutations and genetic diversity among children failing antiretroviral treatment in five healthcare facilities in Benin, West Africa
Source: PLoS One. 2025 Jan 29;20(1):e0317882. doi: 10.1371/journal.pone.0317882 (PMC11778783; doi:10.1371/journal.pone.0317882)
Supplement: S1 Questionnaire — (DOCX) [file pone.0317882.s002.docx]

SURVEY SHEET:

No.……………

**I- IDENTIFICATION:**

I-1-Patient number /______ /

I-2- Age: /______ /

I-3- Gender: 1= M /___ /; 2 = F: /___ /

I-4 - HIV type: /_____/

I-5- Date of inclusion on D0: /______ /

I-6- Weight at inclusion: /______ /

**II- THERAPEUTIC REGIMEN:**

II-1- Child’s ARV protocol

(Check the right answer)

1=ABC+3TC+DTG: /____ /

2=ABC+3TC+LPV/r /____ /

3=ABC+3TC+EFV: /____ /

4=AZT+3TC+DTG: /____ /

5=AZT+3TC+EFV /____ /

6= AZT+3TC+LPV/r: /____ /

7=TDF+ 3TC+LPV/r: /____ /

8=TDF+3TC+EFV /____ /

9=TDF+3TC+DTG: /____ /

II-2- Change of therapeutic line

1 = yes /____ / ; 2 = no /____ /

If yes, specify the new Regimen(s)

Regimen 1: /_________________________ /

Regimen 2: /_________________________ /

Regimen 3: /________________________ /

**III- IMMUNOLOGICAL RESULTS:**

TCD4 value (Cellules/µl):

Initial TCD4: /______ / ; M...:/_______ / ; M...: /_______ / ; M...: /_______ / ; M...: /______/

**IV- VIROLOGICAL RESULTS:**

Viral load (VL): normal (undetectable <40 copies/ml)

- CV (copies/ml) CV (log)

- M1: /________________________ / /________________________ /

- M...: /________________________ / /________________________ /

- M...: /________________________ / /________________________ /

- M...:/________________________ / /________________________ /

- M...: /______________________ / /________________________ /

**V- TAKING THE TREATMENT :**

VI-1- Compliance with treatment:

-Yes /_____ / ; No /_____ /

If no, why ?

1-by forgetting

2-dose error

3-drug sharing

4-taking medication in excess

5-voluntary reduction in the number of doses or tablets

VI- 2 - Therapeutic education of the patient: (1-Good 2-Bad):

1/___ / 2 /___ /

**VI- GENOTYPING**

• NNRTI-mutations

- /________________________ / /________________________ /

- /________________________ / /________________________ /

- /______________________ / /________________________ /

INTI-mutations

- /________________________ / /________________________ /

- /________________________ / /________________________ /

- /______________________ / /________________________ /

IP-mutations

- /________________________ / /________________________ /

- /________________________ / /________________________ /

- /______________________ / /________________________ /

INSTI-mutations

- /________________________ / /________________________ /

- /________________________ / /________________________ /

- /______________________ / /________________________ /
